# Supplementary material for: Exploring the principles behind antibiotics with limited resistance
Source: Nat Commun. 2025 Feb 21;16:1842. doi: 10.1038/s41467-025-56934-3 (PMC11845477; doi:10.1038/s41467-025-56934-3)
Supplement: Supplementary file 1 — Supplementary Information [file 41467_2025_56934_MOESM1_ESM.pdf]

Supplementary information for

Exploring the principles behind antibiotics with limited resistance

Elvin Maharramov, Márton Simon Czikkely, Petra Szili, Zoltán Farkas, Gábor Grézal, Lejla Daruka, Eszter Kurkó, Léna Mészáros, Andreea Daraba, Terézia Kovács, Bence Bognár, Szilvia Juhász, Balázs Papp, Viktória Lázár, Csaba Pál

Corresponding author: cpal@brc.hu

The file includes:

Supplementary Figures 1-8

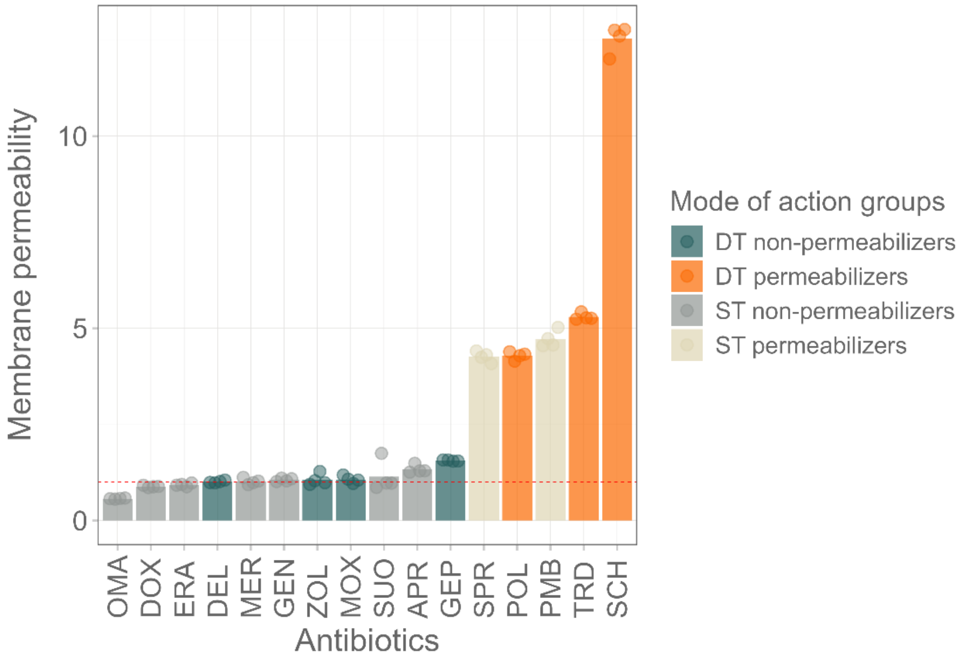

Supplementary Figure 1. Antibiotic induced membrane permeabilization. Outer membrane permeabilization induced by antibiotics was detected using a fluorescent probe (1-N-phenylnaphthylamine, *i.e.,* NPN) uptake (four biological replicates per treatment). *E. coli* ATCC25922 cells were exposed to different antibiotic treatments (at a concentration of 4×MIC) and the intracellular accumulation of the probe was normalised to that of the untreated control. The fluorescence intensity of NPN gradually increases due to the permeabilization of the bacterial outer membrane by SCH79797 (SCH), tridecaptin M152-P3 (TRD), POL7306 (POL), SPR206 (SPR) and polymyxin B (PMB). The dashed line indicates the mean of untreated control. For antibiotic abbreviations, see Table 1. Source data are provided as a Source Data file.

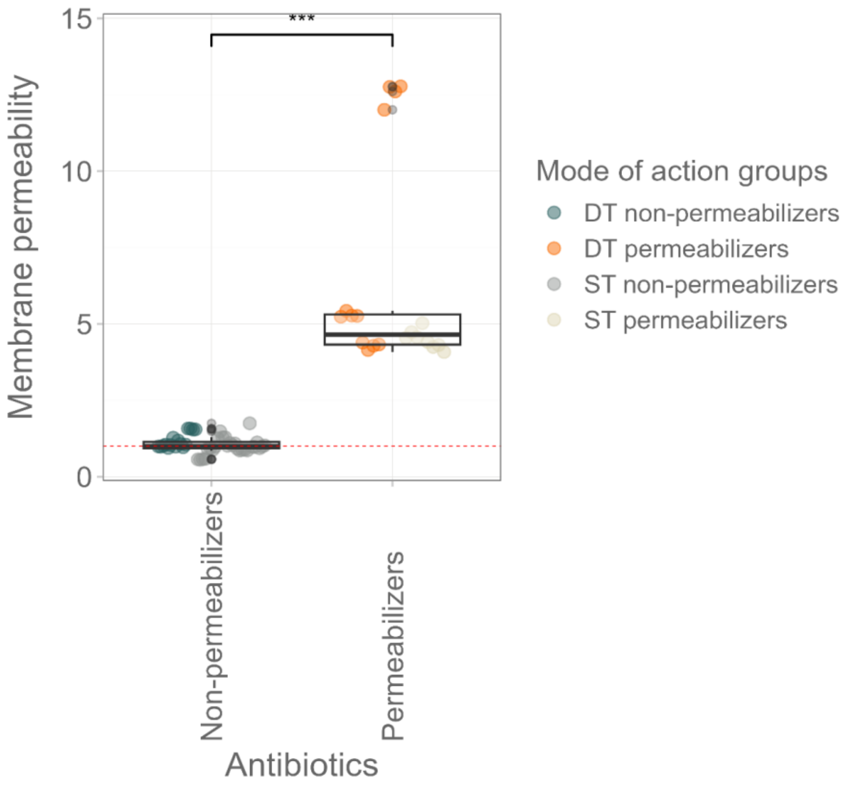

Supplementary Figure 2. Antibiotic induced membrane permeabilization. It was detected by a fluorescent probe (1-N-phenylnaphthylamine, *i.e.*, NPN) uptake (four biological replicates per treatment). *E. coli* ATCC25922 cells were exposed to different antibiotic treatments (at a concentration of 4×MIC) and the intracellular accumulation of the probe was normalized to that of the untreated control. Boxplots show the median, first and third quartiles, with whiskers showing the 5th and 95th percentiles. To assess significant difference between the two groups (non-permeabilizers n = 44; permeabilizers n = 20), two-sided Wilcoxon rank-sum test was used (*** indicates $p = 1.02 \times 10^{-16}$). The dashed red line indicates the mean of untreated control. For antibiotic categories, see Table 1. Source data are provided as a Source Data file.

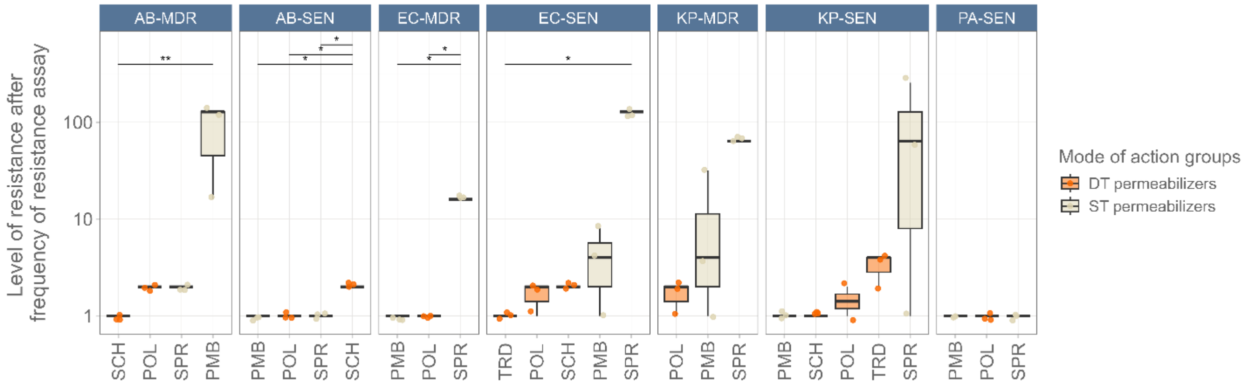

Supplementary Figure 3. Resistance levels following the frequency of resistance assay across bacterial strains. The level of resistance was measured as the fold change in relative MIC between evolved and ancestral strains. Data are grouped by bacterial strain (*A. baumannii* (AB), *E. coli* (EC), *K. pneumoniae* (KP), and *P. aeruginosa* (PA)) and categorized as either multidrug-resistant (MDR) or sensitive (SEN). Each data point represents a laboratory evolved line, and boxplots show the median, first and third quartiles, with whiskers indicating the 5th and 95th percentiles. DT permeabilizers generally resulted in lower or equal resistance levels compared to ST permeabilizers. Statistical analysis was performed using two-sided Dunn's post-hoc test with Benjamini-Hochberg correction for multiple comparisons (**/ * indicates p < 0.01/ 0.05). For detailed information on antibiotic and bacterial species, see Supplementary Table 1. For mode of action groups, refer to Table 1. Source data are provided as a Source Data file.

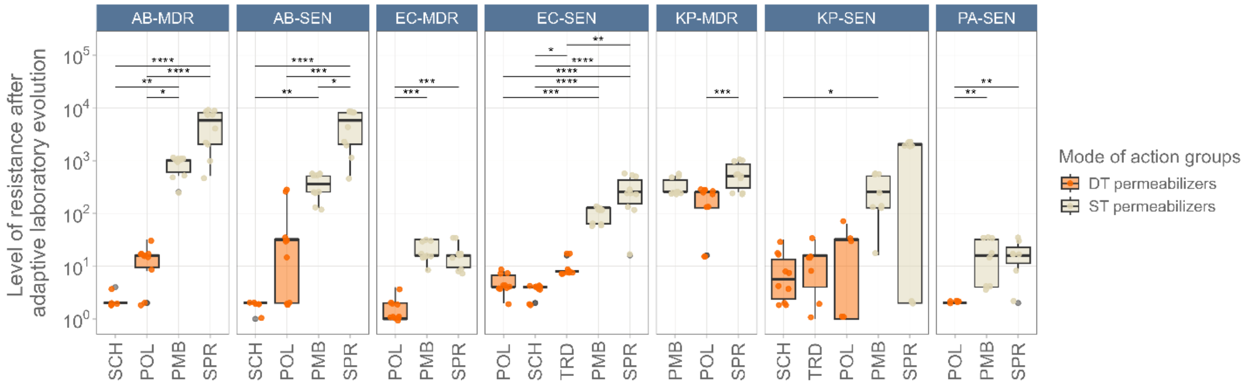

Supplementary Figure 4. Resistance levels following adaptive laboratory evolution across bacterial strains. The level of resistance was measured as the fold change in relative MIC between evolved and ancestral strains after ~60 days of adaptive laboratory evolution. Data are grouped by bacterial strain (*A. baumannii* (AB), *E. coli* (EC), *K. pneumoniae* (KP), and *P. aeruginosa* (PA)) and categorized as either multidrug-resistant (MDR) or sensitive (SEN). Each data point represents a laboratory evolved line, and boxplots show the median, first and third quartiles, with whiskers indicating the 5th and 95th percentiles. DT permeabilizers resulted in lower resistance levels compared to ST permeabilizers, though significant strain-specific variations were observed. Statistical analysis was performed using two-sided Dunn's post-hoc test with Benjamini-Hochberg correction for multiple comparisons (****/ ***/ **/ * indicates p < 0.0001/ 0.001/ 0.01/ 0.05). For detailed information on antibiotic and bacterial species, see Supplementary Table 1. For mode of action groups, refer to Table 1. Source data are provided as a Source Data file.

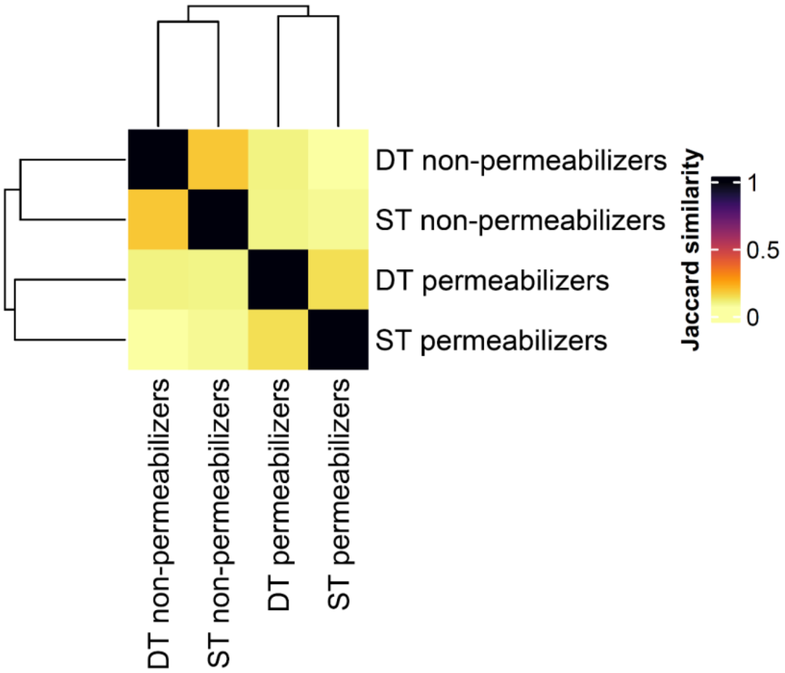

Supplementary Figure 5. Heatmap of mutational profile similarity between mode of action groups. The heatmap shows the mutational profile similarity among all mode of action groups. The Jaccard similarity score was used to calculate the overlap in genes mutated during the course of adaptive laboratory evolution in response to different modes of action groups. The colour gradient from yellow to black represents the range of similarity scores, with yellow indicating no similarity (0), and black representing complete similarity (1). Antibiotic clustering was based on calculating Euclidean distance of mean Jaccard scores and using the complete hierarchical clustering method. Antibiotic clustering was based on calculating Euclidean distance of mean Jaccard scores and using the complete hierarchical clustering method. Source data are provided as a Source Data file.

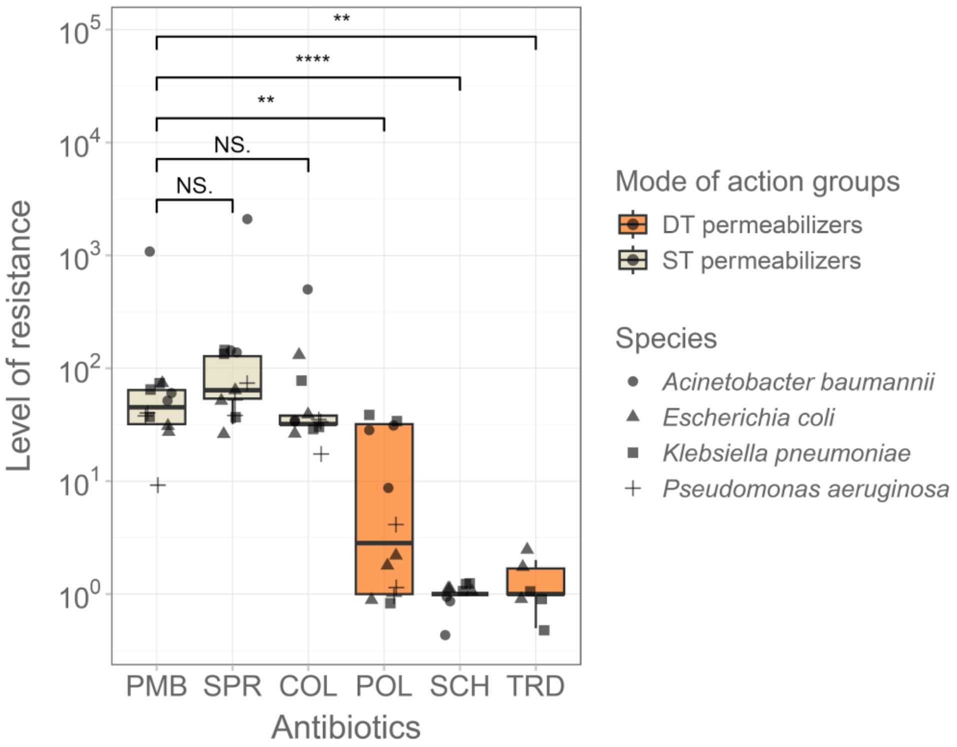

Supplementary Figure 6. Susceptibility of Polymyxin B resistant lines to other ST and DT permeabilizers. The boxplot shows the level of resistance (*i.e.,* relative MIC values calculated by dividing the MIC of the evolved line by that of the corresponding ancestor) of 12 polymyxin B resistant lines to ST and DT permeabilizer antibiotics used in this study. Each data point represents a distinct adaptive laboratory evolved polymyxin B resistant line. Boxplots show the median, first and third quartiles, with whiskers showing the 5th and 95th percentiles. *P* values were calculated using Dunn's post-hoc test with Benjamini-Hochberg correction (NS indicates $p > 0.05$, ** / **** indicates $p < 0.01$ / 0.0001, respectively). The resistance level of polymyxin B resistant lines to polymyxin B (PMB), colistin (COL) and SPR206 (SPR) is comparable to each other. For detailed information on antibiotic and bacterial strain abbreviations, see Supplementary Table 1. Source data are provided as a Source Data file.

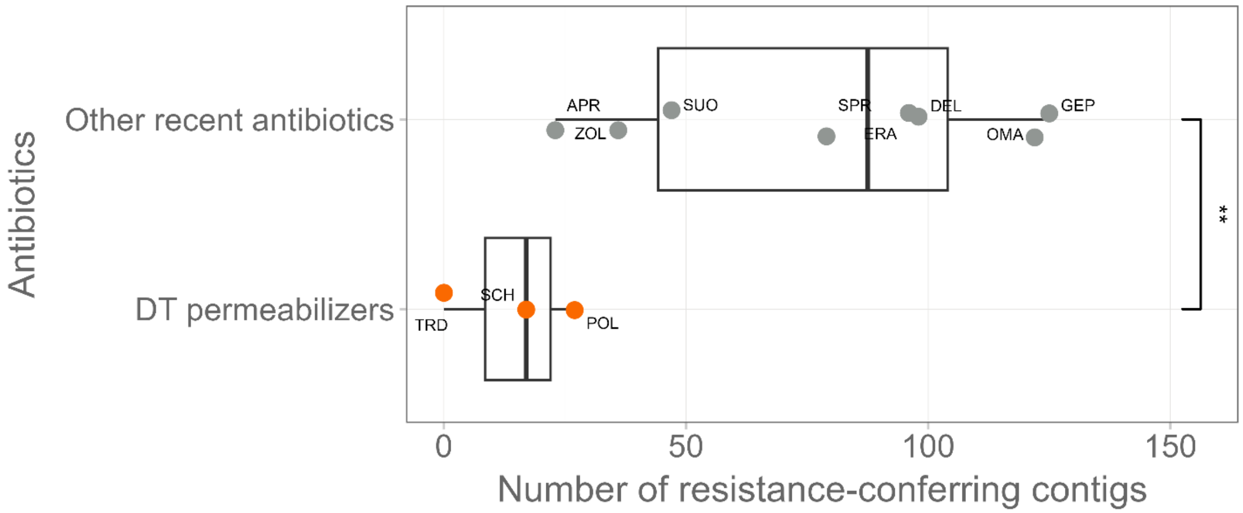

Supplementary Figure 7. **Resistance-conferring contigs in functional metagenomics screens against antibiotics currently in development.** The analysis focuses on new antimicrobial compounds which have been introduced into clinical practice recently (after 2017) or are currently in development (i.e., 'recent' antibiotics[28]). The boxplot shows the distribution of resistance-conferring contigs identified against DT-permeabilizers and other "recent" antibiotics. Each point represents the number of contig for a given antibiotic, and boxplots show the median, first and third quartiles, with whiskers indicating the 5th and 95th percentiles. DT permeabilizers (n = 3) show significantly lower numbers of resistance-conferring contigs compared to other recently developed antibiotics (n = 8). Statistical significance was assessed using two-sided Student's t-test (** indicates p = 0.0031). Source data are provided as a Source Data file.

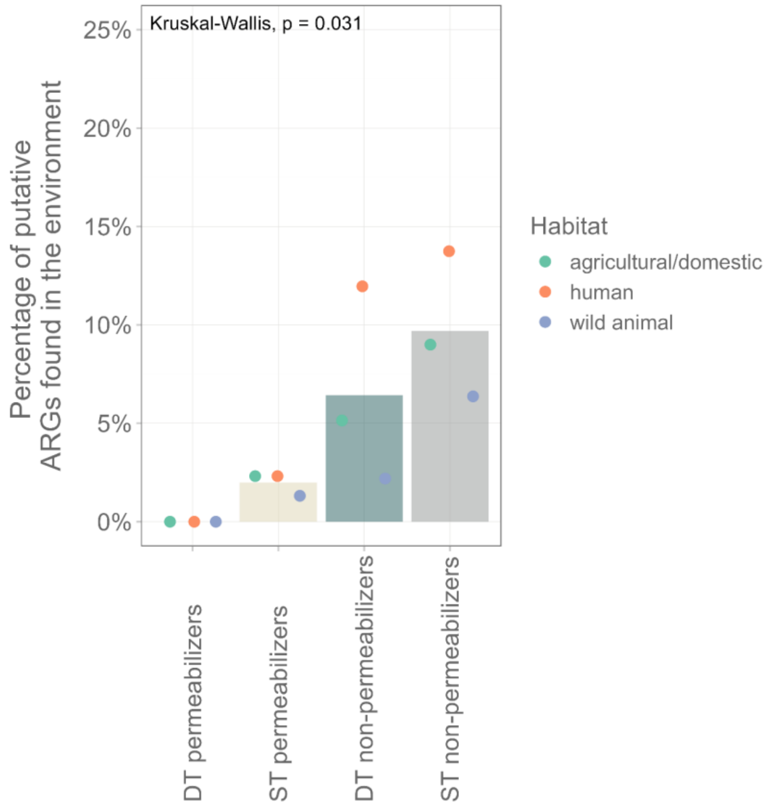

Supplementary Figure 8. Prevalence of putative ARGs in natural *E. coli* genomes across different habitats. The barplot illustrates the percentages of *E. coli* genomes from various natural habitats containing putative antibiotic resistance genes (ARGs). The bars represent the mean percentages of putative ARGs among habitats that provide resistance to each group of antibiotics. Individual data points reflect mean percentages of putative ARGs across antibiotics per habitat. Statistical analysis using a Kruskal-Wallis rank sum test (n = 12, chi-squared = 8.8992, df = 3, p = 0.031) indicated significant heterogeneity in the prevalence of ARGs conferring resistance among groups of antibiotics across different habitats. For grouping based on modes of action, see Table 1. Source data are provided as a Source Data file.
