# Supplementary material for: Exploring the principles behind antibiotics with limited resistance
Source: Nat Commun. 2025 Feb 21;16:1842. doi: 10.1038/s41467-025-56934-3 (PMC11845477; doi:10.1038/s41467-025-56934-3)
Supplement: Supplementary file 3 — Description of Additional Supplementary Files [file 41467_2025_56934_MOESM3_ESM.pdf]

Description of the Supplementary Data files

File name: **Supplementary Data 1.**
Description: Information about antibiotics and bacterial strains used in this study.

File name: **Supplementary Data 2.**
Description: Mutations identified in whole-genome sequenced antibiotic-adapted lines.

File name: **Supplementary Data 3.**
Description: Genes that reduce antibiotic susceptibility upon overexpression (ASKA overexpression library).

File name: **Supplementary Data 4.**
Description: Functional metagenomic data, including information on libraries, DNA contigs and ORFs.

File name: **Supplementary Data 5.**
Description: Killing kinetics of antibiotics.
