# Supplementary material for: Exploring the principles behind antibiotics with limited resistance
Source: Nat Commun. 2025 Feb 21;16:1842. doi: 10.1038/s41467-025-56934-3 (PMC11845477; doi:10.1038/s41467-025-56934-3)
Supplement: Supplementary file 9 — Reporting Summary [file 41467_2025_56934_MOESM9_ESM.pdf]

nature portfolio

Corresponding author(s): Dr. Csaba Pál

Last updated by author(s): Jan 15, 2025

Reporting Summary

Nature Portfolio wishes to improve the reproducibility of the work that we publish. This form provides structure for consistency and transparency in reporting. For further information on Nature Portfolio policies, see our Editorial Policies and the Editorial Policy Checklist.

Statistics

For all statistical analyses, confirm that the following items are present in the figure legend, table legend, main text, or Methods section.

n/a	Confirmed	
☐	☒	The exact sample size (*n*) for each experimental group/condition, given as a discrete number and unit of measurement
☐	☒	A statement on whether measurements were taken from distinct samples or whether the same sample was measured repeatedly
☐	☒	The statistical test(s) used AND whether they are one- or two-sided<br>*Only common tests should be described solely by name; describe more complex techniques in the Methods section.*
☒	☐	A description of all covariates tested
☐	☒	A description of any assumptions or corrections, such as tests of normality and adjustment for multiple comparisons
☐	☒	A full description of the statistical parameters including central tendency (e.g. means) or other basic estimates (e.g. regression coefficient) AND variation (e.g. standard deviation) or associated estimates of uncertainty (e.g. confidence intervals)
☐	☒	For null hypothesis testing, the test statistic (e.g. *F*, *t*, *r*) with confidence intervals, effect sizes, degrees of freedom and *P* value noted<br>*Give P values as exact values whenever suitable.*
☒	☐	For Bayesian analysis, information on the choice of priors and Markov chain Monte Carlo settings
☒	☐	For hierarchical and complex designs, identification of the appropriate level for tests and full reporting of outcomes
☐	☒	Estimates of effect sizes (e.g. Cohen's *d*, Pearson's *r*), indicating how they were calculated

Our web collection on statistics for biologists contains articles on many of the points above.

Software and code

Policy information about availability of computer code

Data collection	Data was obtained from laboratory experiments or in indicated cases from our earlier study (Daruka et al., Nature Microbiology, 2025).
Data analysis	Biotek Gen5 Data Analysis Software; R (4.3.2), MATLAB

For manuscripts utilizing custom algorithms or software that are central to the research but not yet described in published literature, software must be made available to editors and reviewers. We strongly encourage code deposition in a community repository (e.g. GitHub). See the Nature Portfolio guidelines for submitting code & software for further information.

Data

Policy information about availability of data

All manuscripts must include a data availability statement. This statement should provide the following information, where applicable:

- Accession codes, unique identifiers, or web links for publicly available datasets
- A description of any restrictions on data availability
- For clinical datasets or third party data, please ensure that the statement adheres to our policy

The outer membrane permeabilization, gene overexpression, cross resistance, AcrAB-TolC, killing kinetics data generated in this study and MIC fold change data after laboratory evolution used in this study are provided in the Supplementary Information/Source Data file.

The Illumina reads for the gene overexpression experiments generated in this study have been deposited in the European Nucleotide Archive (ENA) database under

accession code PRJEB80327 [https://www.ebi.ac.uk/ena/browser/view/PRJEB80327].

The Illumina reads and Nanopore contigs for whole genome sequencing and functional metagenomic screens used in this study are available in the European Nucleotide Archive (ENA) database under accession code PRJEB63210 [https://www.ebi.ac.uk/ena/browser/view/PRJEB63210].

Research involving human participants, their data, or biological material

Policy information about studies with human participants or human data. See also policy information about sex, gender (identity/presentation), and sexual orientation and race, ethnicity and racism.

Reporting on sex and gender	5 male, 5 female
Reporting on race, ethnicity, or other socially relevant groupings	N/A
Population characteristics	Human stool samples were obtained from ten healthy unrelated individuals, The five male and 5 female participants were in the age range of 26-42 years.
Recruitment	Volunteer participants were selected on the basis of strict criteria that (1) they did not take any antibiotics for at least oneyr before sample donation and (2) they are in a good health. These requirements are standard in the field and secure a bias-free comparison of the antibiotic resistomes in the healthy human gut microbiome. Informed consent was obtained from all participants.
Ethics oversight	Permission for the faecal sample collection was obtained from the Human Investigation Review Board of Albert Szent-Györgyi Clinical Centre, University of Szeged (registered under 72/2019-SZTE).

Note that full information on the approval of the study protocol must also be provided in the manuscript.

Field-specific reporting

Please select the one below that is the best fit for your research. If you are not sure, read the appropriate sections before making your selection.

[x] Life sciences [ ] Behavioural & social sciences [ ] Ecological, evolutionary & environmental sciences

For a reference copy of the document with all sections, see nature.com/documents/nr-reporting-summary-flat.pdf

Life sciences study design

All studies must disclose on these points even when the disclosure is negative.

Sample size	Throughout the work, we chose sample sizes that are in agreement with previously published protocols. Sample sizes are always indicated in the corresponding Methods section or figure legends. The studied antibiotics represent a diverse set of antibiotic classes. The number of antimicrobials investigated met with the standards of previously published studies in the field of antibiotic resistance research (e.g. DOI: 10.1126/scitransmed.3009940, DOI: 10.1093/jac/dkw381).<br>In functional metagenomics, the number of tested treatments, library sizes (2–6 million clones per environment) and number of DNA sources (10 individuals, 6 polluted sites and 68 bacterial species) met with the standards of previously published studies in the field of antibiotic resistance research (DOI: 10.1126/science.1176950, DOI: 10.1038/nature13377). The large number of resistant DNA fragments obtained with antibiotic selection confirmed that our sample size was large enough for the presented study.
Data exclusions	No data was excluded.
Replication	Biological replicates were used to handle random errors. All experimental measurements were repeated independently at least twice involving at least 3 technical replicates each. All attempts at replication were successful.
Randomization	Samples were not allocated into experimental groups.
Blinding	Randomization is not relevant to this study, because the data collection and analysis were not biased by investigators.

Reporting for specific materials, systems and methods

We require information from authors about some types of materials, experimental systems and methods used in many studies. Here, indicate whether each material, system or method listed is relevant to your study. If you are not sure if a list item applies to your research, read the appropriate section before selecting a response.

Materials & experimental systems

n/a	Involved in the study
☒	☐ Antibodies
☒	☐ Eukaryotic cell lines
☒	☐ Palaeontology and archaeology
☒	☐ Animals and other organisms
☒	☐ Clinical data
☒	☐ Dual use research of concern
☒	☐ Plants

Methods

n/a	Involved in the study
☒	☐ ChIP-seq
☒	☐ Flow cytometry
☒	☐ MRI-based neuroimaging

Plants

Seed stocks	*Report on the source of all seed stocks or other plant material used. If applicable, state the seed stock centre and catalogue number. If plant specimens were collected from the field, describe the collection location, date and sampling procedures.*
Novel plant genotypes	*Describe the methods by which all novel plant genotypes were produced. This includes those generated by transgenic approaches, gene editing, chemical/radiation-based mutagenesis and hybridization. For transgenic lines, describe the transformation method, the number of independent lines analyzed and the generation upon which experiments were performed. For gene-edited lines, describe the editor used, the endogenous sequence targeted for editing, the targeting guide RNA sequence (if applicable) and how the editor was applied.*
Authentication	*Describe any authentication procedures for each seed stock used or novel genotype generated. Describe any experiments used to assess the effect of a mutation and, where applicable, how potential secondary effects (e.g. second site T-DNA insertions, mosiacism, off-target gene editing) were examined.*
